# AMADAR: a python-based package for large scale prediction of Diels–Alder transition state geometries and IRC path analysis

**DOI:** 10.1186/s13321-022-00618-3

**Published:** 2022-06-15

**Authors:** Bienfait K. Isamura, Kevin A. Lobb

**Affiliations:** 1 Department of Chemistry, Rhodes University, Makhanda, 6140 South Africa; 2 Research Unit in BioInformatics (RUBi), Rhodes University, Makhanda, 6140 South Africa

**Keywords:** Diels–Alder reaction, RDKit, Reaction force analysis, Finite difference approach, Hellman–Feynman force

## Abstract

Predicting transition state geometries is one of the most challenging tasks in computational chemistry, which often requires expert-based knowledge and permanent human intervention. This short communication reports technical details and preliminary results of a python-based tool (AMADAR) designed to generate any Diels–Alder (DA) transition state geometry (TS) and analyze determined IRC paths in a (quasi-)automated fashion, given the product SMILES. Two modules of the package are devoted to performing, from IRC paths, reaction force analyses (RFA) and atomic (fragment) decompositions of the reaction force F and reaction force constant $\kappa$. The performance of the protocol has been assessed using a dataset of 2000 DA cycloadducts retrieved from the ZINC database. The sequential location of the corresponding TSs was achieved with a success rate of 95%. RFA plots confirmed the reaction force constant $\kappa$ to be a good indicator of the (non)synchronicity of the associated DA reactions. Moreover, the atomic decomposition of $\kappa$ allows for the rationalization of the (a)synchronicity of each DA reaction in terms of contributions stemming from pairs of interacting atoms. The source code of the AMADAR tool is available on GitHub [CMCDD/AMADAR(github.com)] and can be used directly with minor customizations, mostly regarding the local working environment of the user.

**Graphical Abstract:**

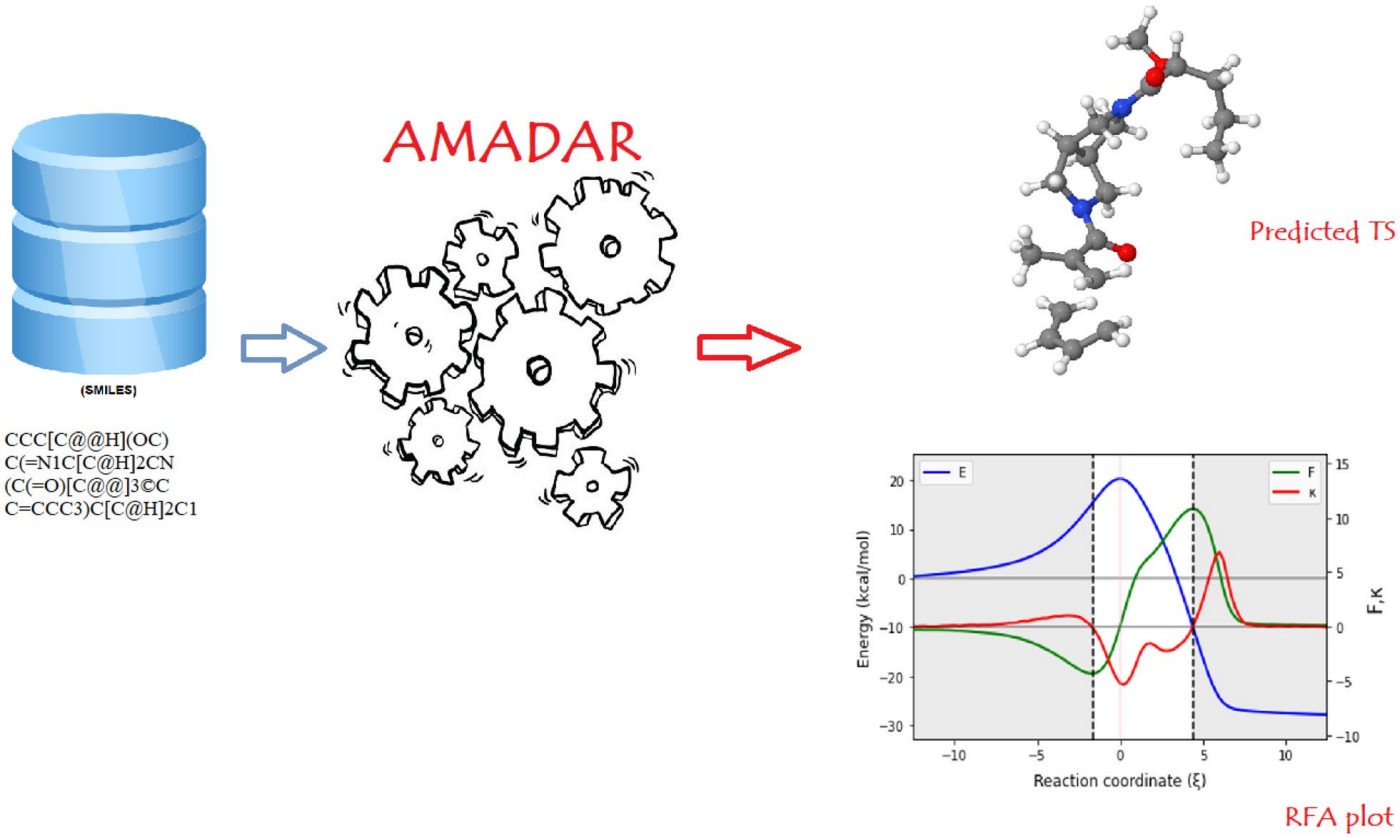

**Supplementary Information:**

The online version contains supplementary material available at 10.1186/s13321-022-00618-3.

## Introduction

In the framework of transition state theory [1], chemical reactions are assumed to proceed through transient configurations named transition states (TSs), which in turn determine the rate of the process. TSs are first-order saddle points on the potential energy surface (PES) suspended at the top of the lowest-energy route joining two local minima corresponding to the reactant and product states [1]. While local minima can be readily attained by descending the steepest route from a panoply of starting points on the PES, locating TSs demands that the search is initiated from a good starting (guess) geometry that is structurally very similar to the actual TS[2]. Unfortunately, guessing such a structure requires expert-based knowledge and consistent human intervention. In addition, the chances of success drop drastically if a large number of TSs have to be generated based on the same guess geometry; because, even for similar reactions, the best initial guesses may be significantly different.

Recently, deep learning (DL) approaches emerged as a potential solution to this problem. These approaches rely on a variety of sophisticated multilayer neural networks that are able to learn hidden features within a dataset and use the accumulated knowledge to make reliable predictions on unseen data [3]. Although sound and promising, DL tools are computationally very demanding and always require huge amounts of good quality data to train the models before any prediction can be made. For instance, Pattanaik and coworkers needed 6800 isomerization reactions to train their graph neural network before testing its predictive power on a reduced set of 850 systems [4]. Moreover, some DL pipelines must be fed with optimized geometries of both the reactants and products [5], which turns out to be a drawback in case these structures have to be first obtained at a high computational level.

On the other hand, the Diels–Alder (DA) reaction is one of the most important reactions in organic chemistry, which has found many applications such as in the total synthesis of natural products [6] and in polymer chemistry [7]. Since its discovery in 1928 [8], the DA reaction has been widely investigated and the debate around its mechanism is still very enthusiastic [9]. Particularly, several computational studies are revisiting the mechanism of the DA reaction using new reactivity paradigms, including, without being restricted to, the reaction force analysis [10], the activation strain model [11], and bond evolution theory [12]. However, in order to perform these analyses, there is the requirement that TSs be first predicted and the reaction path be determined.

Therefore, driven by the need to contribute to the challenge of TS geometries prediction as well as the understanding of the mechanism of the DA reaction, we have built the AMADAR program (Automated workflow for Mechanistic Analysis of Diels–Alder Reactions). In comparison with DL approaches, the AMADAR tool does not involve any training step, works with any number of reactions (even one system suffices) and uses only SMILES strings of the cycloadducts as inputs. It is capable of generating an unlimited number of Diels–Alder (DA) TS geometries, before carrying out subsequent analyses based on the intrinsic reaction coordinate (IRC) paths. Key features of AMADAR include its ability to handle particular cases such as intramolecular reactions and situations resulting in competing paths. The code is also highly customizable. The source code of the AMADAR program is provided with appropriate documentation detailing the functioning of the program. It is written in a user-friendly, efficient way, that should allow intermediate python programmers with some knowledge in computational chemistry to easily customize, where necessary. The AMADAR package is focused on the large community of researchers working on the DA reaction.

**Fig. 1:**
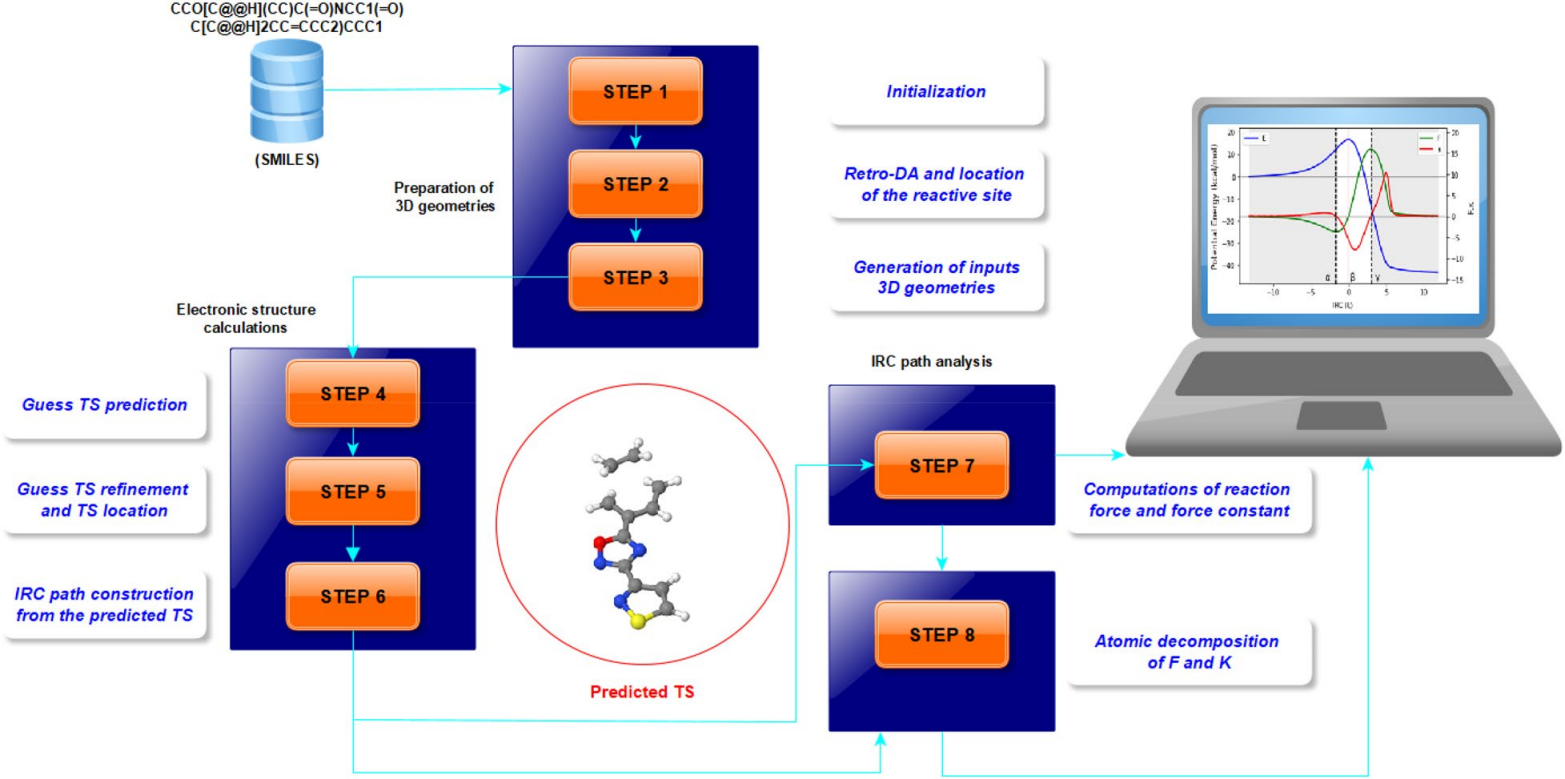
Simplified scheme of the AMADAR algorithm

In the present communication, we report technical details of the algorithm behind the AMADAR tool and present some preliminary results. Table 1 gives a short description of the most important modules. Figure 1 schematizes the AMADAR’s algorithm, which consists of 8 steps divided into three main phases: the preparation of 3D geometries, the electronic structure calculations, and the IRC path analysis. These steps are detailed below.

**Table 1:** Description of the most important modules

Modules	Description	Dependencies	classes (methods)	Length
IRC	Builds input files for IRC calculations and analyzes outputs files	–	2 (51)	2127 lines
RFA	Carries out numerical derivations from IRC paths data; and returns 2D plots of V, F, and K along the IRC path	–	5 (48)	1780 lines
TS	Prepares input files for the refinement of guess TS, and analyzes the outputs	–	1 (36)	1770 lines
Geom_3D	Converts *mol* objects into 3D geometries, and prepares input files for electronic structure calculations	Retro-DA	0 (5)	335 lines
Retro_DA	Realizes the retro-DA transformation of the cycloadducts (CA), and identifies the reactive site for each pathway to the (CA)	–	0 (16)	416 lines
RFD	Carries out the atomic (fragment) decomposition of the reaction force (constant)	IRC	2(20)	525 lines
Guess	Analyzes outputs of the redundant coordinate optimization (pseudo-guesses) and builds inputs for the guess TS	–	2 (27)	817 lines

## Requirements

The usage of the AMADAR program on a computing platform requires a python 3 environment with access to the RDKit [13] toolkit. This is because the initial steps of the algorithm rely on RDKit. In terms of the specific usage with respect to the use of Gaussian 09, the user has to define in the main configuration file (da.ini) all the environment variables giving access to this software. The Gaussian 09 software has been considered as the default, but several other programs could have been used in place (Psi4 [14], Gamess [15], etc.). If other electronic structure software is required for use, the user will need to modify methods that generate input files or methods which analyze outputs (Fig. 1).

## The algorithm

### Step 1

The program is launched from the__*init.py* script from a local RDKit environment. This script accesses SMILES strings of the cycloadducts saved in the SMILES.txt file, and reads user input from the main configuration file (da.ini). This main configuration file contains flags that must be set according to the type of jobs the user will like to carry out. The description and usage of the keywords (flags) in the da.ini file are provided in Additional file 1 (Additional file 3: Table S1).

### Step 2

The SMILES strings accessed are converted into *mol* objects, which are then used to locate and keep track of the reactive sites (RS). As such, an ordered list of atomic indices (ListAtomsInt) is returned, in which four atoms originate from the diene and the two others from the dienophile. In case the cycloadduct has more than one cyclohexene substructure, a 2 × n shaped list is returned, with n the number of cyclohexene substructures. This results in competing paths that are treated separately.

### Step 3

At this step, the 3D geometry of the cycloadducts in Cartesian coordinates are obtained using a sequential procedure, including the embedding of its *mol* object and the optimization of the returned conformer using the UFF force-field [16]. UFF is a broadly applicable force field that contains parameters for almost all atoms of the periodic table. This guarantees no error is returned when studying a system with such uncommon atoms like actinides due to inexistent force field parameters. Moreover, since UFF is a non-reactive force field, the topology of the system under investigation is kept intact during this conformation search, preventing any bond cleavage or formation. In case of failure, the procedure is repeated, this time looking for more conformers (up to 60) and increasing the number of runs (up to 2000). The selected conformer is used as input in the constrained optimization towards the pseudo-guess of the TS (step4).

### Step 4

A constrained optimization in internal coordinates coerces the cycloadducts to adopt a symmetric two-fragment configuration where two pairs of terminal C atoms from the diene and dienophile are separated by 2.15 Å. Note that the positions of these C atoms are retrieved from the ListAtomsInt obtained at step 2. The default separation distance of 2.15 Å can be modified by the user. For this, they have to simultaneously edit the ini.da configuration file and the *Gen_gjf_file_ts()* method of the *Geom_3D.py* module. Using default settings, this optimization returns 16 successive configurations of the system, of which the highest energy structure (a 2 fragment structure for intermolecular DA reactions) corresponds to the pseudo-guess TS. The latter is isolated, then cleaned up at the same level of theory using the TS single-ended Berny algorithm [17]. This gives rise to the guess-TS. The PM6 semi-empirical method has been found to perform well at this step.

### Step 5

**Fig. 2:**
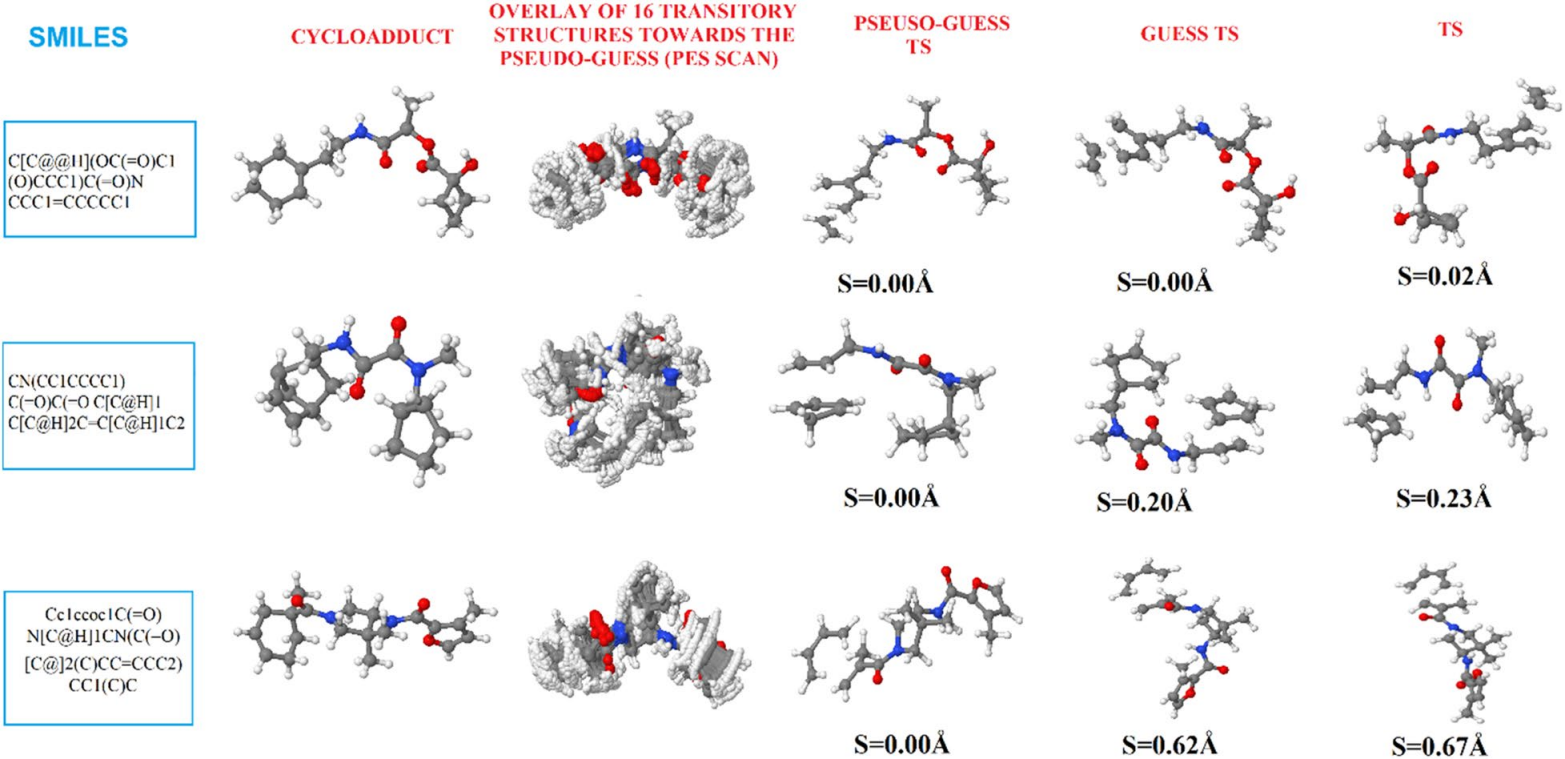
Illustration of the process towards the prediction of TSs by the AMADAR program from SMILES strings of cycloadducts. Synchronicity indexes (S) are given at the different steps

For each system, a new TS calculation is performed to refine the previous guess structure at a user-defined quantum mechanics level of theory. This step is followed by a vibrational check to make sure the predicted stationary point is a real TS. This check is meant to assure that the returned TS has only one imaginary frequency. For this, we examine (extract and count) the normal vibrational modes of the system. Only structures with a unique negative (imaginary) frequency are retained as actual TSs. Rejected stationary points are automatically copied to an appropriate folder named ERROR_FILES. Steps 3–5 are illustrated in Fig. 2 for a small set of three randomly selected systems. Additional file 3: Fig. S1 depicts (optimized) geometries of the associated reactants (diene and dienophile), whose SMILES strings were first generated by applying the *process_retro_Diels_Alder()* function of the *retro_DA()* module to the cycloadduct SMILES, before being sequentially optimized at the PM6 and B3LYP/6-31G(d) levels respectively (Additional file 2).

### Step 6

Once the TS has been located, the IRC path can be determined. Details about the theory level or the number of IRC points are defined by the user in the da.ini file. We have observed that at least 60 points per IRC direction from the TS are sufficient to obtain a good IRC path (for mid-size systems with heteroatoms) linking the reactants to the cycloadducts through the TS. The geometries of the reactants and cycloadducts can be optimized alongside that of the TS (during steps 2–4) if the RC_FLAG in the da.ini is set to 1 (Table 1).

After the IRC calculations, a separate script named *myIRCAnalyzer.py* may be executed to perform *r*eaction force analyses (RFA, step7) and atomic decompositions of the reaction force and reaction force constant (step 8) for specific reactions. Details about the system to analyze or the atoms to consider in the decomposition must be given in a separate configuration file (analysis.ini). The description and usage of all keywords (and sections) found in the analysis.ini configuration files are provided in the Additional file 3: Table S2.

### Step 7

A special module (*RFA)* has been integrated to the package for executing all the calculations related to the reaction force analysis. Details about the RFA paradigm can be found here [18].Two important quantities of the theory are the reaction force F and reaction force constant $\kappa$, which are defined using Eqs. 1 and 2, where E is the system’s energy along the IRC path $\xi$. Torro-Labé and his co-workers have provided strong evidence showing that F and $\kappa$ can help gain insight into the mechanism of several reactions [19].

1 $${F}_{\xi }=-\frac{dE}{d\xi },$$

2 $${\kappa }_{\xi }=\frac{{d}^{2}E}{d{\xi }^{2}}=-\frac{dF}{d\xi }.$$

F and $\kappa$ are numerically calculated at each point of the IRC path using the finite difference approach. Technically, the average value of the forward and the backward derivatives at each given point is used as a good estimation of the exact derivative, except for the first and last points of the IRC path. Any attempt to run this analysis will be ignored if the RFA_FLAG in the da.ini file has not been set to 1.

### Step 8

As demonstrated by Jędrzejewski et al. [20], the reaction force F and force constant $\kappa$ can be decomposed into atomic contributions by introducing the Hellman–Feynman [21] forces acting on each nucleus in the standard definition of F and $\kappa$ (Eqs. 3–4).

3 $${F}_{\xi }=-\frac{dE}{d\xi }=-\sum_{A\in M}\frac{\partial E}{\partial {R}_{A}} \frac{\partial {R}_{A}}{\partial \xi }=\sum_{A\in M}{F}_{A}\frac{\partial {R}_{A}}{\partial \xi }=\sum_{A}{F}_{A}\left(\xi \right),$$

4 $${\kappa }_{\xi }=-\frac{{dF}_{\xi }}{d\xi }=-\sum_{A\in M}\frac{d}{d\xi }\left[{F}_{A}\frac{d{R}_{A}}{d\xi }\right]=\sum_{A}{\kappa }_{A}\left(\xi \right).$$

Furthermore, ${\kappa }_{\xi }$ can be split into two components originating from the atoms and bonds of the molecule (Eq. 5) [20].

5 $${\kappa }_{\xi }=\sum_{A}^{N}{\kappa }_{AA}(\xi )+2\sum_{A}^{N}\sum_{B<A}^{N}{\kappa }_{AB}(\xi )={\kappa }_{\xi }^{atoms}+{\kappa }_{\xi }^{bonds},$$

where the sums run over all the atoms in the molecule.

**Fig. 3:**
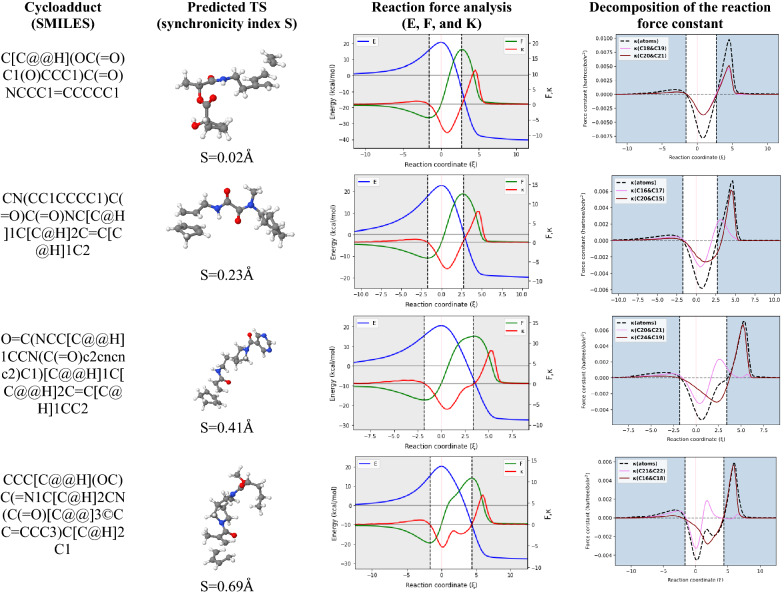
Illustration of key outputs of the AMADAR program: S (Synchronicity index in Å); Reaction force analysis (E, F, in kcal, kcal/$\xi$ and kcal/$\xi$^2^ respectively); Atomic decomposition of the reaction force constant ($\kappa$ in Hartree/bohr^2^): the violet and maroon curves indicate contributions from the two pairs of interacting atoms from the diene and dienophile. The dashed line gives the ${\kappa }_{\xi }^{atoms}$ component of $\kappa$

We have also incorporated in the AMADAR package a module called *RFD*, which implements Eqs. 3–5 in the case of DA reactions. To perform the series of decomposition analyses available in the module, the RFD_FLAG in the da.ini file must be set to 1 before running the myIRCAnalyzer.py script (Fig. 3).

## Preliminary results

To test the performance of the AMADAR tool, a set of 2000 potential Diels–Alder cycloadducts was extracted from the ZINC database (SMILES provided in the Additional file 1 and 4). These compounds were identified by the presence of at least one cyclohexene substructure. The PM6 and B3LYP/6-31G(d) levels were considered for the generation of the guess TS and its refinement respectively. The generation of TSs was successful at ~ 95%, consisting of 1912 TSs located. The remaining 5% of unsuccessful calculations were due to basis set inconsistencies and unmet convergence criteria. Basis set errors were returned for all the systems containing an iodine atom [which does not have a 6-31G(d) basis set], while some very large systems could not achieve their convergence. A separate module is being developed to systematically address these issues. This module will be integrated into the next release of the package. Further, about 150 of the predicted TSs were used for determining the IRC path with a step size of 0.8 (amu)^1/2^Bohr. These systems were chosen to cover a wide range of synchronicity indices (S), measured as the difference of length between the two emerging C–C bonds at the TS. Note that synchronous reactions are those having S values close to 0, while asynchronous ones have higher values. Figure 3 illustrates some of the results obtained for five of the systems studied.

In line with previous findings [22], the reaction force constant is found to be a good indicator of the synchronicity of DA reactions. For synchronous reactions for example, $\kappa$ shows only one minimum in the TS region, while there are one and a shoulder to two minima in the same region for moderate to very asynchronous reactions respectively (Fig. 3). The last column of Fig. 3 suggests that the global synchronicity of DA reactions can be rationalized in terms of contributions of the two pairs of interacting C atoms (from the diene and dienophile) to the reaction force constant $\kappa$. In addition, the position of their respective global maximum seems to indicate the moment when the corresponding C–C bonds achieve their formation. Finally, the position of the global minimum of $\kappa$ with regards to that of the classical TS can be explained in terms of the interplay between the two previous elementary processes, one tending to shift it to the right and the other to the left.

## Concluding remarks

We have presented technical details and preliminary results of the AMADAR package. The latter is expected to be helpful to the broad community of researchers working on the mechanism of DA reactions. A detailed study is being conducted on the large dataset obtained upon the application of the AMADAR tool to 2000 likely DA cycloadducts. This in-depth study aims at understanding common features of the mechanism of the DA reaction through the light of the reaction force analysis and the decomposition of F and $\kappa$. AMADAR is currently limited to homo-DA reactions, but there is work in progress in our group to make possible the investigation of several types of hetero-DA reactions.

## Supplementary Information

**Additional file 1. **AMADAR package.

**Additional file 2. **Predicted TS geometries of 1912 DA reactions with imaginary frequency.

**Additional file 3. **Description and usage of configuration files.

**Additional file 4. **SMILES of potential Diels-Alder adducts.

## Data Availability

The AMADAR program is available from this GitHub repository [CMCDD/AMADAR(github.com)].
