# Supplementary figures and images for: AMADAR: a python-based package for large scale prediction of Diels–Alder transition state geometries and IRC path analysis
Source: J Cheminform. 2022 Jun 15;14:39. doi: 10.1186/s13321-022-00618-3 (PMC9202188; doi:10.1186/s13321-022-00618-3)

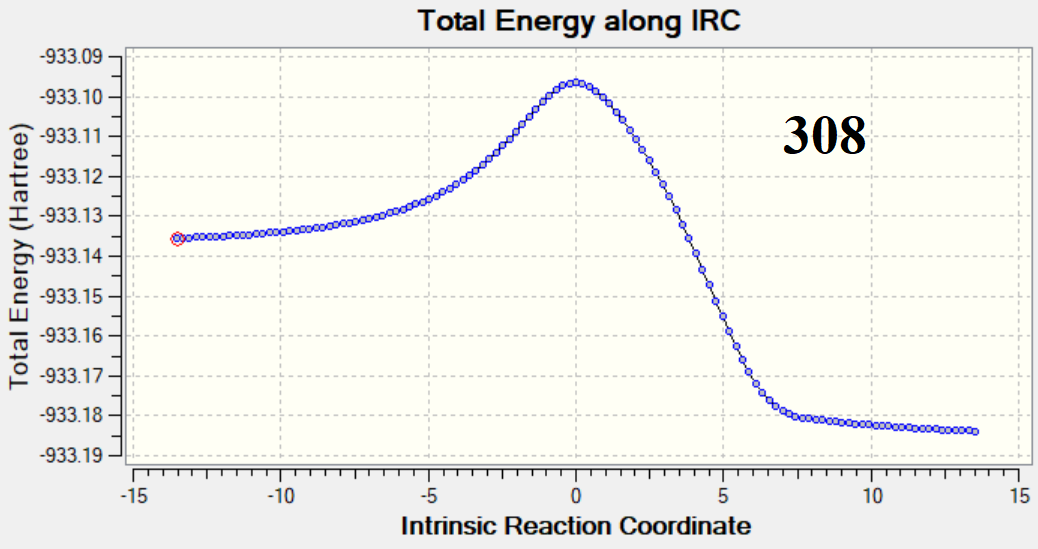

Supplement: Supplementary file 1 — Additional file 1. AMADAR package. [file 13321_2022_618_MOESM1_ESM.zip › AMADAR-main/example2/IRC308.PNG]

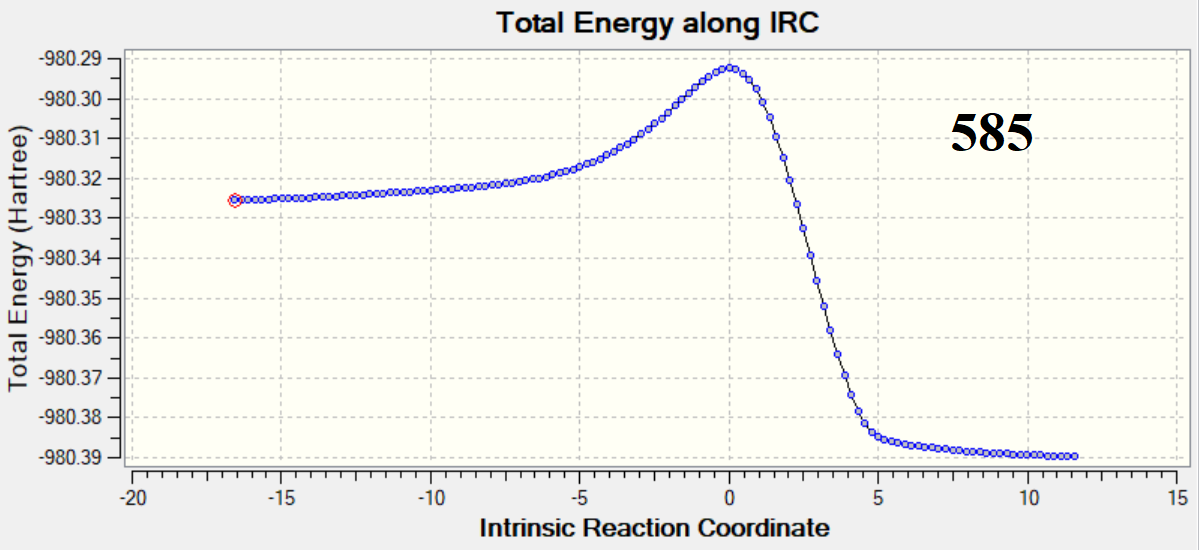

Supplement: Supplementary file 1 — Additional file 1. AMADAR package. [file 13321_2022_618_MOESM1_ESM.zip › AMADAR-main/example2/IRC585.PNG]

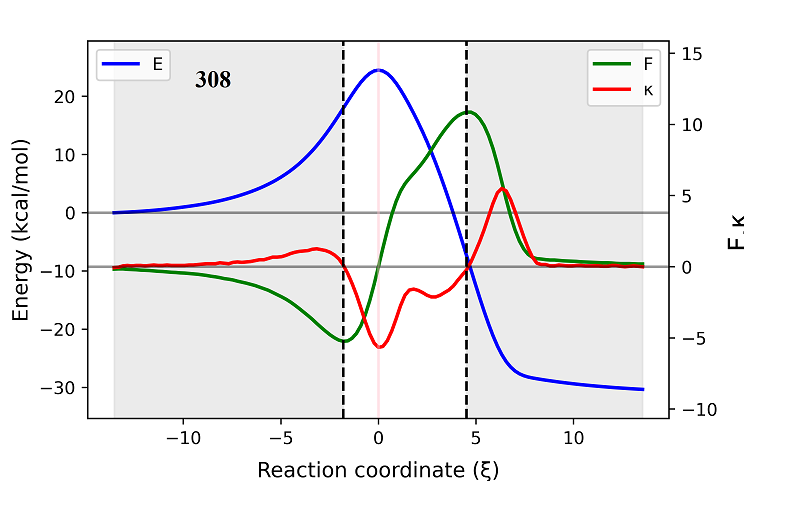

Supplement: Supplementary file 1 — Additional file 1. AMADAR package. [file 13321_2022_618_MOESM1_ESM.zip › AMADAR-main/example3/rfaRx308p1.png]

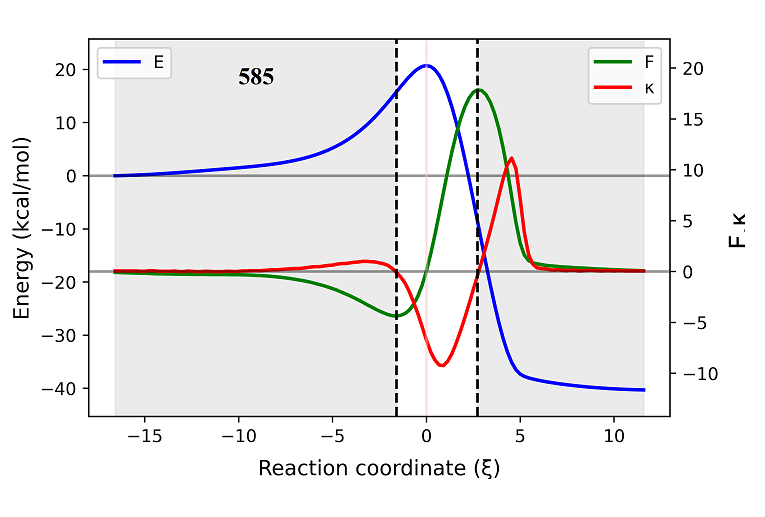

Supplement: Supplementary file 1 — Additional file 1. AMADAR package. [file 13321_2022_618_MOESM1_ESM.zip › AMADAR-main/example3/rfaRx585p1.png]

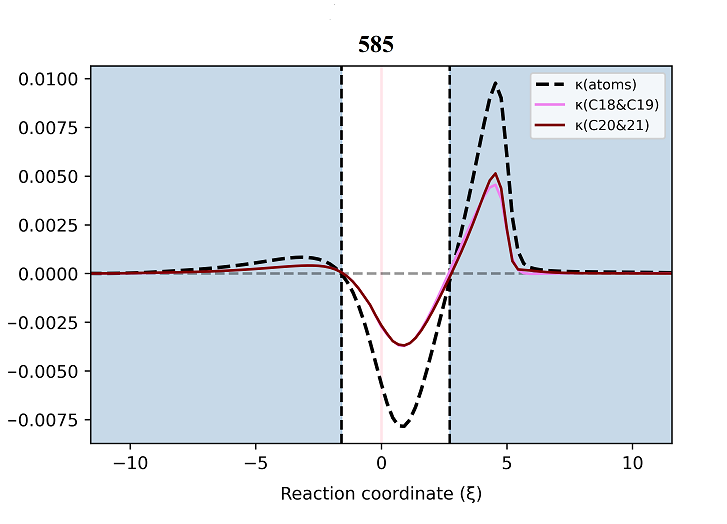

Supplement: Supplementary file 1 — Additional file 1. AMADAR package. [file 13321_2022_618_MOESM1_ESM.zip › AMADAR-main/example4/rfcfdRx585p1.PNG]

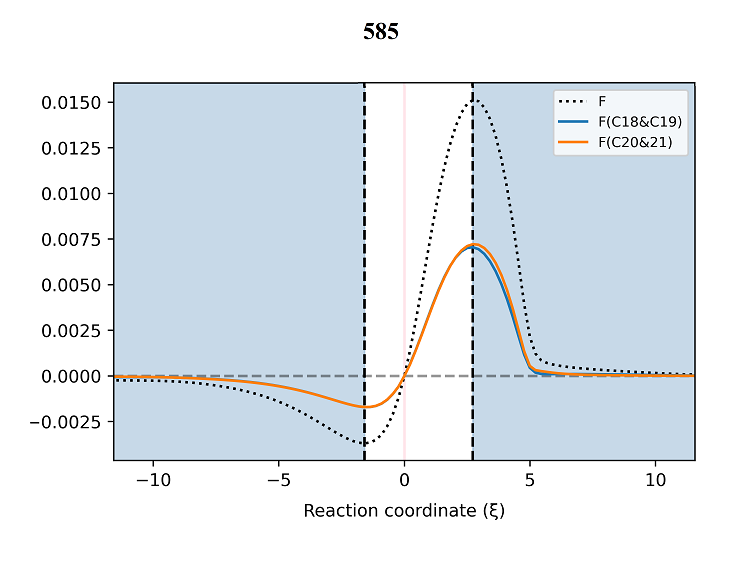

Supplement: Supplementary file 1 — Additional file 1. AMADAR package. [file 13321_2022_618_MOESM1_ESM.zip › AMADAR-main/example4/rffdRx585p1.PNG]

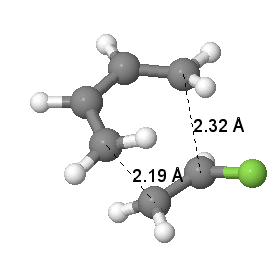

Supplement: Supplementary file 1 — Additional file 1. AMADAR package. [file 13321_2022_618_MOESM1_ESM.zip › AMADAR-main/graphics/TS.png]

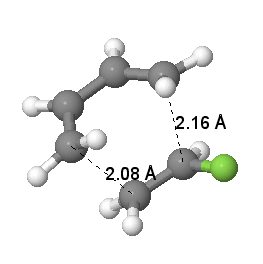

Supplement: Supplementary file 1 — Additional file 1. AMADAR package. [file 13321_2022_618_MOESM1_ESM.zip › AMADAR-main/graphics/guess.png]

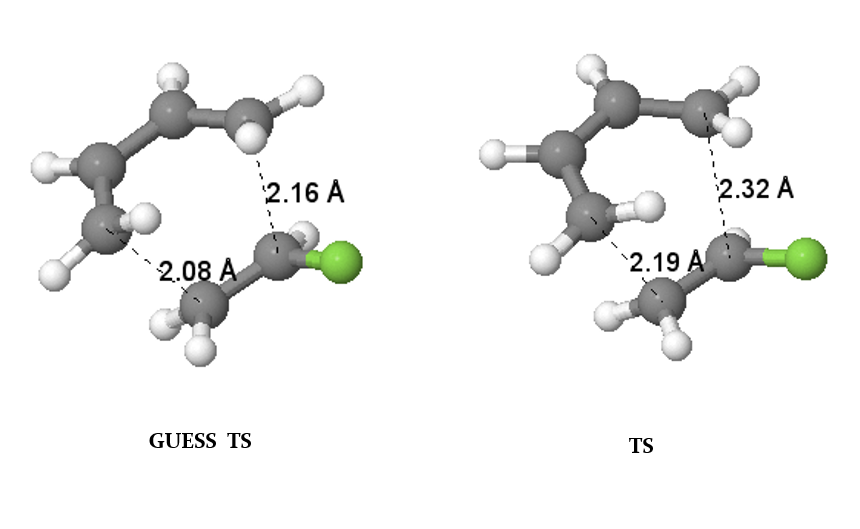

Supplement: Supplementary file 1 — Additional file 1. AMADAR package. [file 13321_2022_618_MOESM1_ESM.zip › AMADAR-main/graphics/guess_and_TS.PNG]

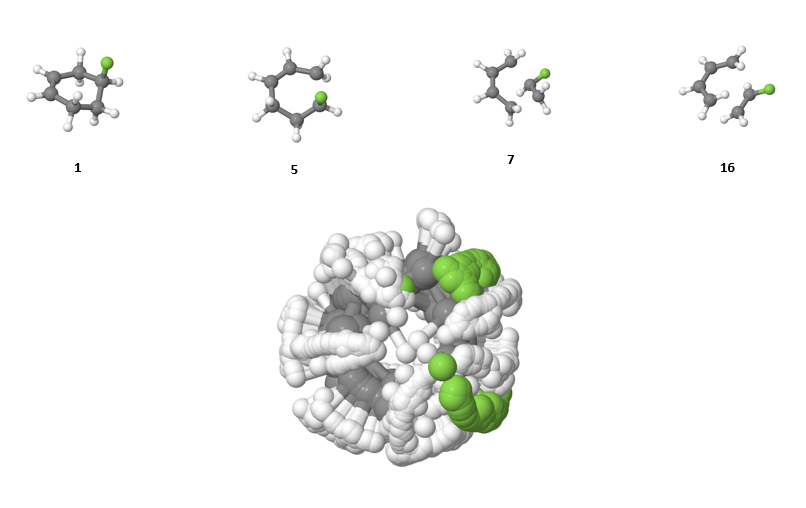

Supplement: Supplementary file 1 — Additional file 1. AMADAR package. [file 13321_2022_618_MOESM1_ESM.zip › AMADAR-main/graphics/pguessTS.png]
