# Supplementary material for: AMADAR: a python-based package for large scale prediction of Diels–Alder transition state geometries and IRC path analysis
Source: J Cheminform. 2022 Jun 15;14:39. doi: 10.1186/s13321-022-00618-3 (PMC9202188; doi:10.1186/s13321-022-00618-3)
Supplement: Supplementary file 3 — Additional file 3. Description and usage of configuration files. [file 13321_2022_618_MOESM3_ESM.docx]

AMADAR: a python-based package for large scale prediction of Diels-Alder transition state geometries and IRC path analysis

Bienfait K. Isamura ^1^ and Kevin Lobb ^1, 2, *^

^1^ Department of Chemistry, Rhodes University, 6140, Makhanda, South Africa

^2^ Department of Biochemistry and Microbiology, Research Unit in BioInformatics (RUBi), Rhodes University, 6140, Makhanda, South Africa

^*^ Corresponding author

Additional file 3

**Table S1. First configuration file: da.ini**

| Section | Keyword | Description | Usage |
| --- | --- | --- | --- |
| **[job_details]** | NPROCSHARED | number of processors to be used for the jobs | Can have any integer value according to the computer platform |
|  | D_SPLITTING | Final distance (in Å) between the two fragments during the constrained optimization. | Default value = 2.15Å. Should be chosen based on chemical intuition |
|  | CALC_LEVELS_RC | Levels of theory to be used in the optimization of reactants and cycloadducts. Two levels must be given, separated by a semi-colon. | The defaults are PM6 and b3lyp/6-31G(d). |
|  | CALC_LEVELS_TS | Levels of theory to be used in the optimization of reactants and cycloadducts. Three levels must be given, separated by a semi-colon. | The first level is used in the constrained optimization of the cycloadduct towards the pseudo-guess TS. The second level is for the refinement of the pseudo-guess into a guess TS. It is better using the same level as the 1st. The third level is needed for the final refinement of the guess-TS to the targeted QM accuracy. The defaults are PM6, PM6 and b3lyp/6-31G(d). |
|  | CALC_LEVEL_IRC | The level of theory to be used in IRC calculations. | Default is B3LYP/6-31G(d). This should be the same as the one used to refine the guess into the likely TS. |
|  | NBR_IRC_POINT_PP | Maximum number of points to use in the construction of the IRC path. | Default = 60 is good starting choice for mid-sized molecules, but may be increased or decreased depending on the system. |
|  | IRC_STEP_SIZE | Step-size to consider in the determination of the IRC path. | The default is 8, corresponding to 0.08 (amu) ^0.5^ Bohr |
|  | NBR_PATHS | Nature of IRC path to be constructed. | This keyword can have two states: 1 in case we want to build a unique IRC file or 2 if we want to have 2 separate input files. |
|  | TS_ID_numbers | ID numbers of the reactions for which for which the IRC path has to be generated. These values correspond to the position (line) of the SMILES string in the SMILES.txt file. | These must be integers values separated by commas. This flag can also be set to 'ALL', which means all the TS predicted will be involved in IRC calculations. |
| **[flags]** | SCRATCH | Tells the initialization code whether it should overwrite the existing files in the R, C and TS folders or not. | This flag can only have 1 and 0 values, which tell the code to overwrite or not the existing folders. In case 0 is chosen, then there must already exist files in the R, C and TS folders. |
|  | RC_FLAG | Tells the initialization code whether it should optimize the reactants and cycloadducts geometries or not. | This flag can only have 0 and 1 values. Value 0 urge the code not to optimize the reactants and cycloadducts. |
|  | TS_FLAG | Tells the initialization code whether it should optimize the TS or not. | This flag can only have 0 and 1 values. Value 1 tells the code to optimize the TSs, whereas 0 says the opposite |
|  | IRC_FLAG | Tells the initialization code whether it should run IRC calculations or not. | This flag can only have 0 and 1 values. Value 1 tells the code to run IRC calculations, whereas 0 says the opposite. This will work if and only if there are TS already predicted and saved in the ...TS/GTS/TS folder. |
|  | IRC_GEOMS_CONSTR | Tells the code to generate geometries of the system along the IRC path. | This will work only if there are IRC paths already constructed and saved in the IRC folder. Outputs are saved in the "paths" folder. |
|  | RFA_FLAG | Authorizes or blocks the code to run the reaction force analysis. | This flag can only have 0 and 1 values. Value 1 is for authorizing, o for blocking. |
|  | RFD_FLAG | Authorizes or blocks the atomic resolution of energy derivatives (along the IRC path) to be performed. | This flag can only have 0 and 1 values. Value 1 is for authorizing, o for blocking. |
|  | WBOA_FLAG | Controls the execution of Wiberg bond order analysis based on IRC geometries. It will work only if IRC natural population calculations have already been performed on the IRC geometries. These are saved in the "paths" folder. | This flag can only have 0 and 1 values. Value 1 enables the Wiberg Bond Order analysis to be carried out, while 0 is for the opposite. |

**Table S2. Second configuration file: analysis.ini**

| Section | Keyword | Description | Usage |
| --- | --- | --- | --- |
| **[RFA]** | Unq_RFA | This keyword indicates the list of ID numbers of IRC files to include in the reaction force analysis. Values must be integers, separated by commas. | In case all the files are to be considered, the value "-1" must be used. The value 0 means that no file is to be analysed. |
|  | Multiple_RF | It indicates the list of ID numbers of IRC files to include in the multiple RFA in order to superimpose their reaction force curves. Values must be integers, separated by commas. | The value "-1" is not allowed as the number of overlayable systems must stay limited for efficiency. The value 0 disables the analysis |
|  | Multiple_RE | It indicates the list of ID numbers of IRC files to include in the multiple RFA in order to superimpose their potential energy curves. Values must be integers, separated by commas. | The value "-1" is not allowed as the number of overlayable systems must stay limited for efficiency. The value 0 disables the analysis. |
|  | Multiple_RFC | This keyword indicates the list of ID numbers of IRC files to include in the multiple RFA in order to superimpose their reaction force constant curves. Values must be integers, separated by commas. | The value "-1" is not allowed as the number of overlayable systems must stay limited for efficiency. The value 0 disables the analysis. |
| **[RFD]** | JOB_ID | This keyword indicates the ID number of IRC file to consider for the atomic decomposition of energy derivatives. | The value 0 disables the analysis. |
|  | ATOMS | This keyword gives the list of atomic indexes to involve in the decomposition. Values must be integers, separated by commas. | The value 0 disables the analysis. |
|  | FRAG | This keyword is for the list of fragments to involve in the decomposition. | Fragments are separated by semi-colons, while indexes of atoms from the same fragment are separated by commas. Make sure all the values are integers. The value 0 disables this analysis. |
|  | FRAG_NAMES | Names of the fragments whose indexes of constituting atoms were given in the FRAG keyword. | Names are strings and must be given in the same order as indicated in the FRAG keyword. This section can be left blank or put any value when FRAG=0 because no fragment decomposition will be made. |
| **[IRC_PATHS]** | IRC_ID | ID number of the IRC file for which the geometries have to be extracted and run (single point calculation, with pop analysis) | ID numbers must be integers. |
|  | LEVEL | Level of theory to use for the previous job | Default =B3LYP/6-31G(d) |
|  | WBOA_ID | ID number of the system (IRC file) for which a Wiberg Bond order analysis should be done based on the output of the previous step. | This will work if single point calculations for the geometries extracted from the IRC path of the system of interest was be run and saved in the "path" folder. The value 0 (zero) blocks this module to be executed. |


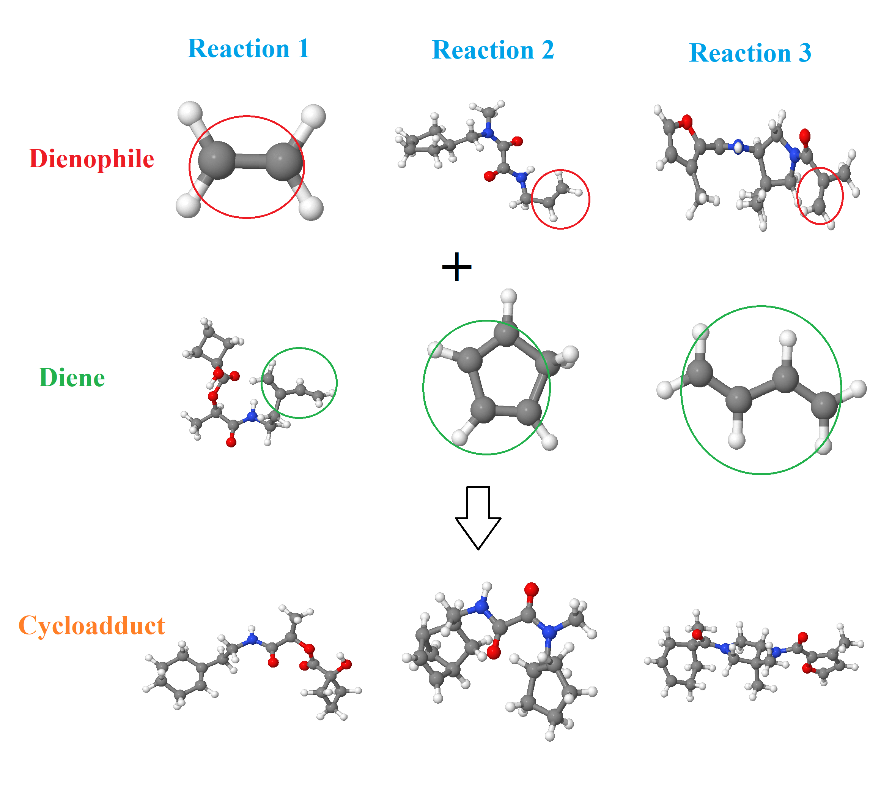


Figure S1. B3LYP/6-31G(d) optimized structures of the reactants and cycloadducts for 3 reactions whose TS generation process is illustrated in Figure 2.
